# Inappropriately low aldosterone concentrations in adults with AIDS-related diarrhoea in Zambia: a study of response to fluid challenge

**DOI:** 10.1186/1756-0500-1-10

**Published:** 2008-04-17

**Authors:** Trevor Kaile, Isaac Zulu, Ruth Lumayi, Neil Ashman, Paul Kelly

**Affiliations:** 1 Tropical Gastroenterology and Nutrition group, University of Zambia School of Medicine, Lusaka, Zambia; 2 Institute of Cell and Molecular Science, Barts & The London School of Medicine, London UK; 3 Renal Unit, Barts and The London NHS Trust, London, UK

## Abstract

**Background:**

Chronic diarrhoea is one of the most debilitating consequences of HIV infection in sub-Saharan Africa and it carries a high mortality rate. We report unexpectedly low concentrations of circulating aldosterone in 12 patients (6 men, 6 women) in the University Teaching Hospital, Lusaka, who all had diarrhoea for over one month. Changes in serum electrolytes, blood pressure, Karnofsky score and serum aldosterone concentration were being monitored during a short study of responses to saline infusion (3 litres/24 h) over 72 hours.

**Findings:**

At baseline, 9/12 (75%) of the patients were hyponatraemic, 10/11 (91%) were hypokalaemic, and 6/12 (50%) had undetectable aldosterone concentrations. Blood pressure and Karnofsky score rose and creatinine concentration fell in response to the infusion.

**Conclusion:**

Circulating aldosterone concentrations were inappropriately low and complicate the profound electrolyte deficiencies resulting from chronic diarrhoea. Management of these deficiencies needs to be more aggressive than is currently practised and consideration should be given to a formal clinical trial of mineralocorticoid replacement in these severely ill patients. If the inappropriately low aldosterone reflects a general adrenal failure, it may explain a considerable proportion of the high mortality seen both before and after initiation of anti-retroviral therapy.

## Findings

### Rationale for the study

Intestinal infection leading to diarrhoea is one of the most disabling and serious manifestations of AIDS. Although anti-retroviral therapy reduces the incidence and severity of diarrhoeal disease in HIV-infection, patients still present to Zambian hospitals with advanced disease, and mortality is still unacceptably high, even after initiating anti-retroviral therapy [1]. In our previous experience, mortality exceeds 20% per month, depending on the specific aetiological infection [2]. There have been many analyses of the spectrum of opportunistic pathogen in AIDS [3-5], but surprisingly little is known about the metabolic consequences of HIV infection in Africans.

It would be anticipated that patients with persistent diarrhoea would have sodium and water depletion and hypokalaemia. During a series of studies of intestinal permeability tests using monosaccharide and disaccharide excretion ratios [6], we noted very low urine outputs in AIDS patients on the point of discharge from hospital. We then undertook fluid challenge studies in these patients, as part of which we assessed electrolyte and mineralocorticoid responses to the fluid challenge. We anticipated that serum aldosterone would be high initially and would fall to normal on successful salt and water repletion, but instead we found inappropriately low aldosterone concentrations.

### Investigations undertaken and methods

Twelve adult patients with diarrhoea of over one month duration, who had all completed initial resuscitation with fluids and were being prepared for discharge, were included in the study. Only one patient per week (chosen at random) was studied due to resource constraints, so this study represents a subset of the patients with chronic diarrhoea admitted to the hospital over the period of the study. A further six patients were eligible for inclusion but were excluded or withdrew for different reasons (see Results). Patients were ineligible for study if there was any evidence of overt cardiac dysfunction or fluid overload (tachycardia, lung crepitations on auscultation, elevated jugular venous pressure, abnormal electrocardiogram). The study was performed in early 2004, just before the advent of expanded access to anti-retroviral drugs in Zambia. Most patients were unaware of their HIV status before the counselling and testing which we carried out. The study was approved by the Research Ethics Committee of the University of Zambia.

Following written informed consent, patients were asked to remain in hospital for the 72 hours of the study. Clinical evaluation included full physical examination (with particular reference to evidence of fluid overload as this was an exclusion criterion), lying and standing blood pressure, weight and height and electrocardiography. The Karnofsky score [7] was used as a measure of global well-being. Body Mass Index (BMI) was calculated as Quetelet's index (kg/m^2^).

At 0800 on the first morning, a blood sample was drawn while the patient was still supine, taken to the laboratory for centrifugation, and aliquots of serum were stored at -80°C. Further blood samples were taken after 24, 48 and 72 hours. Over each 24 hour period, an intravenous infusion of 3 l normal saline was given and the clinical evaluation repeated at 8 hourly intervals. All urine was collected and 24 h sodium excretion estimated.

Analysis of sodium and potassium concentrations in serum was performed on a flame photometer (Corning, Halstead, Essex, UK); glucose and creatinine concentrations were measured on a Cobas Mira multi-analyser (Hoffman-LaRoche, Basel, Switzerland). Aldosterone was assayed in serum samples in London by competitive radioimmunoassay (Diagnostic Products Corporation, Los Angeles, USA); the assay range is 69–3460 pmol/l and precision is approximately 10% (K. Noonan, pers comm.). Effective plasma osmolality (EPO), estimated glomerular filtration rate (eGFR), and total sodium deficit (TSD) were calculated using standard equations [8].

Data are presented as mean with either 95% confidence intervals (CI) or standard deviation (SD). Statistical significance was tested using a paired *t *test on values at the beginning and end of the 72 hour infusion, and a *P *value of < 0.05 regarded as significant.

### Patient characteristics and responses to saline infusion

**Figure 1:**
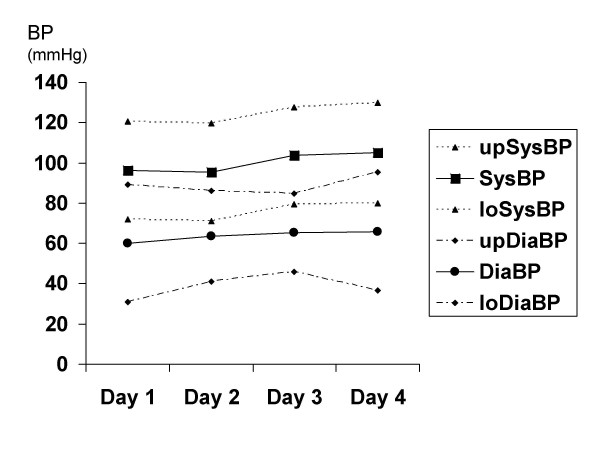
**Changes in blood pressure during saline infusion**. Systolic (SysBP) and diastolic (DiaBP) blood pressure over the 4 days of the trial. Mean values are shown, together with upper (up) and lower (lo) 95% confidence intervals. The rise in systolic BP was significant (p = 0.02) but there was no significant change in diastolic BP.

**Figure 2:**
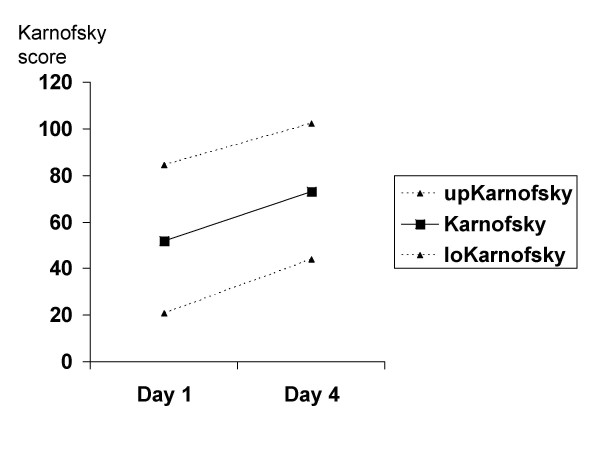
**Changes in Karnofsky score during saline infusion**. Karnofsky score was recorded on days 1 and 4; mean and upper (up) and lower (lo) 95% confidence intervals; the rise was highly significant (p < 0.001).

**Figure 3:**
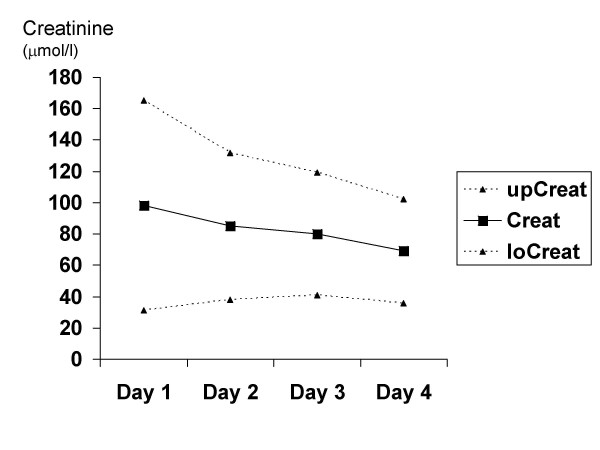
**Changes in creatinine during saline infusion**. Changes in serum creatinine concentration over the course of the 4 days of the fluid challenge; data are presented as mean with upper (up) and lower (lo) 95%CI. The rise was significant (p = 0.04).

We recruited 18 patients into the study, which was conducted in a side ward of a general medical ward in the University Teaching Hospital, Lusaka, from February to April 2004. Of these one had overt renal failure and was excluded, 3 died during the first day and 2 withdrew before the end of the study because of discomfort cause by daily venepuncture. Several of the patients were severely wasted (Table 1). Patients showed variable serum sodium concentrations, with most having moderate and some having profound hyponatraemia (Table 2). These patients tolerated well the large volume of intravenous saline infusion, and in response to this we observed a significant rise in systolic (p = 0.02) but not diastolic (p = 0.29) blood pressure (Fig 1), clinical improvement as reflected in rising Karnofsky score (p < 0.001; Fig 2), and falls in serum creatinine (p = 0.04; Fig 3). Systolic blood pressure rose from a mean of 96 (95%CI 72–121) mmHg at baseline to 105 (80–130) mmHg on day 4. There was no overall rise in serum sodium or potassium concentrations: mean (SD) serum sodium was 123 (12) mmol/l on day 1 and 127 (10) mmol/l on day 4, and serum potassium was 2.8 (1.0) mmol/l on day 1 and 2.7 (0.7) mmol/l on day 4. However, some patients with the most extreme hyponatraemia did show such a rise (for example from 107 to 115 mmol/l and from 114 to 133 mmol/l). All patients demonstrated either hyponatraemia at baseline or a progressive rise in lying blood pressure in response to infusion, and we conclude that all patients had evidence of sodium depletion even though none showed postural hypotension. Further evidence for sodium depletion is that mean urine sodium excretion in the first day was 13 mmol/24 h but that it rose to 38 mmol/24 h after 3 days of saline infusion. In the presence of low aldosterone concentrations this probably signifies that sodium delivery to the distal tubule was very low indeed. There was a mean positive fluid balance of 1.5 litres/24 h, representing a total positive balance of 4.5 litres in 72 hours. Urine outputs in the first 24 hours averaged 25.7 ml/kg in women and 30.2 ml/kg in men.

**Table 1:** Baseline clinical characteristics

ID	Sex	Age (yrs)	HIV status	WHO clinicalHIV stage	BMI (kg/m^2^)	Karnofsky score	BP (lying)	BP (standing)	RR (/min)	Pulse (i/e)
1	F	21	Positive	4	7.8	30	70/40	70/40	26	72/74
2	F	36	Positive	4	11.4	50	100/60	110/70	18	74/74
3	F	25	Positive	4	15.8	50	90/60	100/60	20	80/80
4	F	29	Positive	4	12.0	50	90/60	100/60	24	82/80
5	F	33	Positive	4	21.1	50	110/70	110/80	22	88/88
6	F	25	Positive	4	21.3	50	100/30	110/40	24	88/100
7	M	48	Positive	4	21.8	70	90/60	-	22	80/86
8	M	27	Positive	4	27.3	50	100/60	110/70	28	82/84
9	M	32	Positive	4	22.8	80	120/90	130/90	26	88/90
10	M	31	Positive	4	22.5	50	100/60	110/60	26	90/96
11	M	32	Positive	4	23.4	30	90/60	90/60	28	96/98
12	M	45	Positive	4	20.0	50	100/70	110/70	20	68/70

BMI, body mass index (Quetelet's index); BP, blood pressure; RR respiratory rate; i/e, inspiratory/expiratory.

**Table 2:** Baseline biochemical characteristics

ID	Na^+ ^(mmol/l)	K^+ ^(mmol/l)	Creatinine (μmol/l)	Aldosterone (pmol/l)	EPO (mosmol/kg)	eGFR (ml/min)	TSD (mmol)
1	107	2.4	79	730	215	37	330
2	132	2.3	139	<69	269	29	124
3	136	-	151	628	275	40	86
4	114	2.6	61	3035	232	87	507
5	121	5.9	118	<69	244	60	513
6	111	2.6	72	621	224	94	696
7	143	3.1	71	178	290	93	0
8	141	2.6	67	<69	285	117	0
9	112	2.0	131	545	225	62	1226
10	113	2.7	87	<69	227	88	1102
11	117	2.3	134	<69	250	45	856
12	123	2.1	63	<69	252	96	632

EPO, effective plasma osmolality; eGFR, estimated glomerular filtration rate; TSD, total sodium deficit. One K^+ ^value is missing due to technical failure.

### Inappropriately low circulating aldosterone concentrations

**Figure 4:**
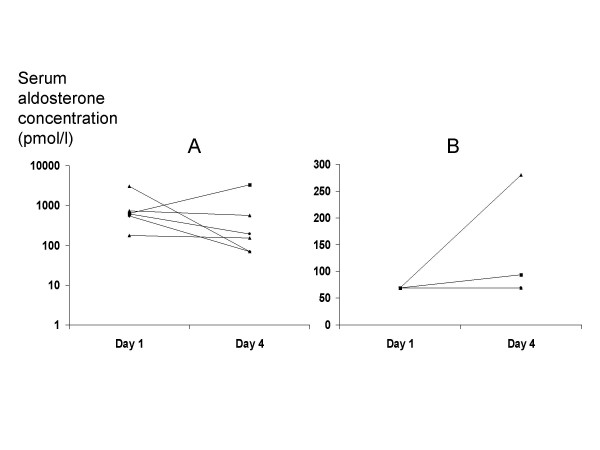
**Changes in aldosterone during saline infusion**. Serum aldosterone concentration before and after fluid challenge in **A **patients with initially normal or high concentrations, and **B **patients with initial concentrations below threshold of assay. In **B**, aldosterone concentrations remained below threshold in 4 patients whose lines overlap and are therefore indistinguishable. Reference range for serum aldosterone in supine patients is 135–400 pmol/l. Note that even 'normal' serum aldosterone would be inappropriately low in patients with sodium depletion.

Initial serum aldosterone concentrations were below the threshold of detection in 6 (50%) of 12 patients. In an additional patient with an apparently 'normal' aldosterone we suspect that the aldosterone was inappropriately low in the face of low initial blood pressure which rose after fluid challenge (Fig 4). In one patient with initially undetectable aldosterone we observed the aldosterone rise into the normal range, but in five it did not. In the patients with initially normal or high aldosterone only one showed a fall into the normal range.

### Interpretation of findings

In this study, performed just before the widespread introduction of anti-retroviral drugs, we found severe deficits of sodium and potassium requiring aggressive therapy. With large volumes of 0.9% saline given intravenously, considerable improvements in well-being and in clinical parameters were observed: average Karnofsky score rose from 50 to 70, systolic blood pressure rose by 9 mmHg, and creatinine fell by around 35%. While we cannot exclude that the rise in Karnofsky score might have been due to other aspects of care, including nursing, the fall in creatinine suggests that renal perfusion had improved markedly due to restoration of vascular and interstitial fluids. The deficits of sodium in these patients appear to be very large and, at least partly due to the chronicity of the primary disease, difficult to correct. Despite the infusion of an average of 1,350 mmol of Na, serum sodium concentrations remained low. We did not test for cortisol or ACTH in the sera which we collected as the amount of serum available was small, but we interpret the inappropriately low serum aldosterone concentrations to signify that mineralocorticoid deficiency should be considered likely in such patients. Supine aldosterone is not the most sensitive test for adrenal failure, and a short Synacthen test might have revealed even more. We cannot say just how common this finding is as our sample is small and we do not know if it is fully representative of all our patients with persistent diarrhoea.

The profound disturbances of electrolytes are difficult to explain fully. The primary disorder, chronic diarrhoea, leads to chronic sodium and potassium loss and total body deficits of both these cations. In otherwise healthy individuals the physiological response to these chronic losses would be activation of the renin-angiotensin-aldosterone cascade which, together with release of vasopressin, would lead to avid renal retention of salt and water, together with potassium loss. Isolated adrenal failure, Addison's disease, would lead to sodium loss and potassium retention. Only one of our patients, number 5 in Table 1, displayed this prototypical biochemical profile at baseline. Most of the other patients had a combination of hyponatraemia and hypokalaemia which seems to indicate that electrolyte loss and low circulating aldosterone concentrations were operating concurrently. In response to a saline challenge the sodium concentrations did not return to normal, suggesting either that the deficit was very substantial or that ongoing losses were continuing, or both. There was no postural hypotension, probably indicating that there was a degree of adaptation to the chronic sodium deficiency and suggesting that ADH was able to some degree to compensate, leading to partial restoration of circulating volume at the expense of hyponatraemia.

### Discussion of findings in the context of available literature

In malnourished children, adrenocortical hormone concentrations in plasma are, in general, appropriately elevated [9], but adults with tuberculosis in Africa frequently have adrenal failure. In a study in Tanzania, adrenal failure was found in 16 of 50 patients with pulmonary tuberculosis, though information on HIV status was not available [10]. To our knowledge no information is available on adrenal dysfunction in HIV infected Africans with persistent diarrhoea, in whom malnutrition can be severe [11]. Adrenal failure in AIDS was described in the 1980s, but it remains an under-diagnosed problem. Adrenal failure in AIDS was recognised first in the 1980s [12] due to cytomegalovirus (CMV) and it has now been recognised as an important complication of HIV infection [13,14]. CMV [15], tuberculosis [10], *Pneumocystis jiroveci*, toxoplasmosis, and lymphoma have all been implicated [16]. Studies in Africa are much more limited, largely due to the lack of availability of reliable endocrine assays. Post-mortem studies in Africa suggest that TB is frequently undiagnosed during life [17] and adrenal tuberculosis is common in Zambian adults with AIDS (V. Mudenda, pers comm.). Recent evidence has indicated that routine tests for adrenal insufficiency may be inadequate and may under-diagnose the condition [18].

We have previously demonstrated that patients with AIDS-related diarrhoea often have severe undernutrition and very high mortality rates [2]. The mortality rate in this study was of great concern. Although based on only 3 deaths, it represents a 17% death rate in one day. We do not think it was due to the intervention as no patient developed fluid overload, but we cannot rule this out. It may be due to the advanced disease which these patients undoubtedly had, particularly severe electrolyte deficiencies and other ions such as magnesium may be important. It is worrying that the patients we recruited were on the point of discharge from hospital, as this would indicate that we have hitherto seriously underestimated the severity of their clinical condition. As patients with AIDS often present to health care workers with late stage disease, we foresee that management of these patients will present a challenge for years to come despite improved access to anti-retroviral therapy. There are very few hospitals in Africa which can perform hormone assays, but we propose that formal controlled trials of mineralocorticoid replacement be set up to find if such a strategy would reduce mortality.

## Abbreviations used

AIDS: Acquired immune deficiency syndrome; HIV: Human immunodeficiency virus; BMI: Body mass index; EPO: Effective plasma osmolality; TBW: Total body water; TSD: Total sodium deficit; GFR: Glomerular filtration rate; CMV: Cytomegalovirus.

## Competing interests

The authors declare that they have no competing interests.

## Authors' contributions

TK, IZ, and PK designed the study, TK, RL and PK carried out the work and all authors prepared and commented on at least some of the data. The manuscript was largely written by TK, NA and PK but all authors contributed discussion and comments.
